# Detecting susceptibility genes for rheumatoid arthritis based on a novel sliding-window approach

**DOI:** 10.1186/1753-6561-3-s7-s14

**Published:** 2009-12-15

**Authors:** Qiuying Sha, Rui Tang, Shuanglin Zhang

**Affiliations:** 1 Department of Mathematical Sciences, Michigan Technological University, 1400 Townsend Drive, Houghton, MI 49931, USA; 2 Mathematical Sciences, Heilongjiang University, Harbin 150080, PR China

## Abstract

With the recent rapid improvements in high-throughout genotyping techniques, researchers are facing a very challenging task of large-scale genetic association analysis, especially at the whole-genome level, without an optimal solution. In this study, we propose a new approach for genetic association analysis based on a variable-sized sliding-window framework. This approach employs principal component analysis to find the optimal window size. Using the bisection algorithm in window size searching, the proposed method tackles the exhaustive computation problem. It is more efficient and effective than currently available approaches. We conduct the genome-wide association study in Genetic Analysis Workshop 16 (GAW16) Problem 1 data using the proposed method. Our method successfully identified several susceptibility genes that have been reported by other researchers and additional candidate genes for follow-up studies.

## Background

With the availability of large-scale genotyping technologies, the cost of genome-wide analyses has been greatly reduced and a boom of large-scale genetic association studies is underway. A sliding-window approach, in which several neighboring single-nucleotide polymorphisms (SNPs) together included in a "window frame", is a popular strategy of multiple allelic association analysis. During the test the window slides across the genome region under study in a stepwise fashion [1-3]. Variable sized sliding-window approaches with variable window sizes decided by the underlying linkage disequilibrium (LD) pattern perform more efficiently in large-scale data analysis. The problem for variable sized sliding-window approaches is how to search the optimal window size with being not only computationally practical but also statistically sufficient to gain higher detection power for both common and rare risk factors.

In this report, based on the variable sized sliding-window frame, we adapt the optimal window size to the local LD pattern by employing principal components (PC) approach. The PC approach is known as a linear projection method that defines a lower-dimensional space and captures the maximum information of the initial data [4]. Each optimal window size is defined by the first few PCs (i.e., 3 or 5) that could explain a main fraction of the total amount (i.e., 90% or 95%) of information in the data.

## Data

In our study, we used the Genetic Analysis Workshop (GAW) 16 Problem 1 data, which is the initial batch of the whole-genome association data for the North American Rheumatoid Arthritis Consortium (NARAC). Data were available for 868 cases and 1194 controls. There are 22 chromosomes with 545,080 SNP-genotype fields from the Illumina 550k chip. To avoid the missing value problem, any subject who had missing values in that window was excluded from the current window. Thus, some subjects may not be in the current window but will still be included in the study in other windows. In this way, we retained the most information we could.

## Methods

### Optimal window size defined by PC analysis

We consider a study with total *M *individuals in a data set and with genotype information denoted by vectors *G*_*i *_= (*g*_*i*1_, *g*_*i*2_,⋯, *g*_*iN*_)^*T *^(*i *= 1,2,⋯, *M*) at *N *SNP loci for the *i*^th ^individual. We code the genotype *g*_*ij *_as 0, 1, or 2 for the number of minor (less frequent) alleles at SNP *j*, *j *= 1,2⋯, *N *of individual *i*. Let *y*_*i *_denote the trait value of individual *i*.

In the sliding-window frame, a window denoted as  is a set of neighboring SNPs {*b*, *b *+ 1, *b *+ 2, ⋯, *b *+ *l *- 1}. A variable sized sliding window which begins with SNP *b*, denoted as Ω^*b*^, is a collection of windows  with *l *ranging from *s *to Γ^*b*^, where *s *and Γ^*b *^are the smallest and largest window sizes.

In this study, we apply PC method to define the optimal window size. The basic idea is that we attempt to find the largest window size in which *c*_0 _proportion of the total information can be explained by the first *k *PCs and *c*_0 _and *k *are predefined criteria. We define this largest window size as the optimal window size. Start with a window  with *l *= *s*= *k *+ 1, so that at least the window length is longer than the number of the important PCs.

Let  denote the sample variance-covariance matrix of genotypic numerical codes in window  and  denote the *j*^th ^largest eigenvalue of  Thus, in window , the total variance in the original dataset explained by the *j*^th ^PC is . Let  as the proportion of the total variability explained by the first *k *PCs. Our main idea of choosing the optimal window size of each sliding window is to find the largest window size in which *c*_0 _proportion of the total variability can be explained by the first *k *PCs among a set of windows Ω^*b*^.

### Bisection method for searching the optimal window size and computational consideration

Using the exhaustive searching method may be computational demanding for determining the optimal window size. We propose to use bisection method. Let *s *and Γ denote the predefined smallest and largest window sizes among a set of windows Ω^*b*^, where b is the starting SNP of the set of windows.

By adapting bisection method, the searching procedure for the optimal window size in Ω^*b *^includes following steps:

Step 1: Let *l *be the middle point of *s *and Γ, that is, *l *= [(*s *= Γ)2/], where [a] is the largest integer that is less than or equal to a.

Step 2: Conduct PC analysis within the window , where a window begins at SNP *b *and has a size *l*.

Step 3: Calculate *C *(the proportion of the total variability explained by the first *k *PCs) for the window . If *C *> *c*_0_, we let *s = l*, that is, we update the smallest window size *s*. Otherwise, we let Γ = *l*, that is, we update the largest window size Γ.

Step 4: Repeat Step 1 to Step 3 until Γ - *s *≤ 1.

In the window , if the proportion of the total variability explained by the first *k *PCs is greater than *c*_0_, the optimal window size will be Γ; otherwise, the optimal window size will be *s*.

Until now, we have not mentioned how to choose the starting SNP *b*. Of course for the first window, *b *= 1. To choose *b *for other windows, the following three methods are typically used. For the *i*^th ^(*i *> 1) window, choose 1) *b = i*; 2) *b *= *n*_*i*_, where *n*_*i *_is the middle SNP of the (*i*-1)^th ^window; 3) *b *= *m*_*i *_+1, where *m*_*i *_is the last SNP of the (*i*-1)^th ^window. In this article, we use the first method to choose the starting SNP *b*.

By using bisection method, our proposed variable length sliding-window method is computationally efficient. Consider a set of windows Ω^*b *^with the smallest window size *s*, largest window size Γ, and starting SNP *b*. The computational complexity to find the optimal window size in Ω^*b *^using the bisection algorithm is Γ^3^log_2_(Γ - *s*). If we have *N *SNPs in total, the computational complexity to find all the optimal window sizes is *N*Γ^3^log_2_(Γ - *s*). In this article, we use Γ = 35 and *s *= 4. Suppose *N *= 500,000 in a genome-wide association study. Then, *N*Γ^3^log_2_(Γ - *s*) <*N*^2^. As pointed out by one of the reviewers, HAPLOVIEW program may be used to find beginning and end of a window. Using HAPLOVIEW, *N*^2 ^of pair-wise *r*^2 ^need to be calculated. To calculate *r*^2^, we need to estimate haplotype frequencies. Theoretically, our proposed method should be computationally more efficient than HAPLOVIEW. In fact, we have done a preliminary simulation study. The results show that the computation time of our proposed method is about a hundred times faster than HAPLOVIEW.

### Score test

After we find the optimal window size for each sliding window, we use the score test statistic based on a logistic model [5] to test for association within each sliding window. Consider , a window beginning at SNP *b *with an optimal window size *l*. Take *b *= 1 as an example for windows that start at the first SNP. Let  denote its first *k *PCs of the *i*^th ^individual, where *i *= 1,2,⋯, *M*. Suppose that the *k *PCs follow a logistic model, then, the score test statistic is given by *T*^2 ^= *U'V*^-1^*U*, where , , , and *M *is the sample size. The statistic *T*^2 ^asymptotically follows a χ^2 ^distribution with *k *degrees of freedom. We select significant windows after adjusting for multiple testing using a Bonferroni correction.

## Result

We applied the proposed approach to GAW16 Problem 1. In our application, we set *s *= 4; Γ = 35; *c*_0 _= 90% and *k *= 3. Originally the dataset contained genotypes at 545,080 SNPs on chromosomes 1 to 22. In our analysis we ended up with 531,501 windows. The size of the windows varied from 4 to 29 SNPs, with the median window size of 7 (see Table 1 for the distribution of the window sizes). After Bonferroni correction, we found 1,155 significant windows. Due to the strong LD among SNPs, many of the significant windows overlapped with the nearby windows. In order to report the result thoroughly, we combined the significant windows with all overlapped windows as one larger window. Thus, we end up with 76 significant larger windows. Due to the limited pages, in Table 2 we only report the top 30 windows after the combination. The order of the windows is according to their most significant sub-windows (the original window before combinations). Our result matches most of the genes reported in recent studies [6-11] and also identify more rheumatoid arthritis (RA) susceptibility genes for follow-up studies.

**Table 1:** The distribution of the window sizes based on 531,501 windows

Window size	Percentage of total windows
4	18.32
5	11.92
6	11.69
7	11.57
8	8.94
9	7.34
10	6.34
11	4.67
12	3.73
13-29	15.48

**Table 2:** Genetic and physical map locations of window region identified using PC-sliding-window analysis based on the Bonferroni correction

Window ID	Chr^a^	Physical location	Genes^b^	CRASG^c^
1	6	30014670, 33187144	*TNF, HLA-A HLA-B, HLA-C*	*TNF, HLA-A HLA-B, HLA-C*
2	1	792429, 1101089	*AGRN, C1orf159, ISG15, SAMD11*	
3	2	172768404, 172807000	*DLX1, DLX2*	*STAT4, ITGAV*
4	12	46666298, 46718200	*COL2A1, LOC728181 LOC728114*	
5	13	113656958, 113861908	*FAM70B, RASA3*	
6	7	154133201, 154241160	*PAXIP1, LOC202781*	
7	17	68283979, 68361160	*SLC39A11*	
8	2	98261543, 98370780	*VWA3B*	*IL1B*
9	13	49336428, 49340230	*KPNA3*	
10	1	2243956, 3359357	*ARHGEF16, PRDM16*	
11	17	66647226, 66750860	*Intergenic 17q24*	
12	16	67482002, 67660490	*TMC07, FLJ12331*	
13	18	75300466, 75314140	*NFATC1*	
14	13	74883232, 74941310	*TBC1D4*	
15	9	123211883, 123248000	*CRB2, MIRN601, DENND1A*	*TRAF1/C5*
16	20	57796484, 57832810		
17	1	15181683, 151846600	*ADAM15, EFNA4*	
18	20	35438689, 35501280	*SRC, RPL7AL4*	
19	22	28164734, 28237820	*RFPL1S, RFPL1, NEFH*	
20	11	64593946, 64661480	*SAC3D1, NAALADL1 CDCA5, ZFPL1, ZHIT2*	
21	11	45207308, 45314100	*SYT13, FLJ41423*	
22	8	20327035, 20435200		
23	5	137614229, 137825000	*GFRA3, CDC25C, FAM53C, JMJD1B*	
24	7	129525353, 129580000	*CPA2, CPA4*	
25	3	134954925, 134966000	*TF, SRPRB*	
26	9	104811123, 104815000	*ABCA1*	
27	12	6924169, 6932652	*ATN1, CL2orf57, PTPN6*	
28	10	10531181, 10542841	*SH3PXD2A*	
29	11	3335218, 3524620	*ZNF195, OR7Z12p*	
30	19	19106771, 19154190	*TMEM16A1, MEF2B*	

^a^Chr, chromosome

^b^We found the significant genes using the NCBI dbSNP database http://www.ncbi.nlm.nih.gov/sites/entrez?db=snp.

^c^The confirmed RA susceptibility genes (CRASG) are shown in the last column if they are within or near our significant region.

## Discussion

As the most exhaustive searching engine in genome-wide association studies, sliding-window approaches are receiving more and more attention recently. Based on the variable sized sliding-window frame, we adapt the optimal window size to the local LD pattern by employing the PC approach. We applied this novel sliding-window approach to the GAW16 RA data and successfully validated nine genes that have been reported by recent studies and also identified new candidate genes for follow-up studies.

Our approach has several advantages. It provides a stable method to choose the window size with the maximum information extraction and it automatically balances degrees of freedom and number of tests, which results in higher power to detect association. It is flexible enough to conduct different association tests within the windows. The method is computational efficient when applied to large-scale data compared with other variable sized sliding-window methods. It requires only genotype data so there is no need to go through any computationally intensive phasing program to account for uncertain haplotype phases.

Further efforts are needed to improve the proposed method, such as determining the optimal *c*_0 _(the proportion of the total variability explained by the top k PCs) and the initial window lengths in the bisection method.

## Conclusion

In this study, we applied our novel genome-wide PC sliding-window approach to detect the association between SNP windows and disease status using GAW16 Problem 1 RA dataset. We validated nine genes which have been identified to be responsible for RA in the literature and discovered more genes and non-gene regions for follow-up studies.

## List of abbreviations used

GAW: Genetic Analysis Workshop; LD: Linkage disequilibrium; NARAC: North American Rheumatoid Arthritis Consortium; PC: Principal components; RA: Rheumatoid arthritis; SNP: Single-nucleotide polymorphism

## Competing interests

The authors declare that they have no competing interests.

## Authors' contributions

QS participated in the design of the study and contributed to the manuscript preparation. RT performed the statistical analysis and wrote the draft of the manuscript. SZ contributed to the design of the study and to the manuscript preparation. All authors read and approved the final manuscript.
